# The Complication of a Retained Broken Distal Interlocking Screw Within a Cephalomedullary Nail During Conversion Hip Arthroplasty: A Case Report

**DOI:** 10.7759/cureus.37075

**Published:** 2023-04-03

**Authors:** Olivia Panchal, Matthew J Schultz, Rex W Lutz, Eric B Smith, Gregory K Deirmengian

**Affiliations:** 1 Orthopaedic Surgery, Texas College of Osteopathic Medicine, Fort Worth, USA; 2 Orthopeadic Surgery, Jefferson Health New Jersey, Stratford, USA; 3 Orthopaedic Surgery, Rothman Orthopaedic Institute, Philadelphia, USA

**Keywords:** midshaft femur fracture and open reduction internal fixation, complication of treatment, femoral nail, total hip athroplasty, hip conversion

## Abstract

In patients who undergo femoral fracture fixation with a cephalomedullary nail, the breakage of one or more of the distal interlocking screws is a well-described phenomenon. The presence of a broken interlocking screw in patients who require the removal of their cephalomedullary nail presents a unique challenge. The broken interlocking screw may be retrieved, or the screw may be retained if it is not engaged within the nail and the nail can safely be removed while leaving the broken screw fragment behind. We report a hip conversion arthroplasty case with a broken interlocking screw where the nail was removed with ease and the broken screw was assumed to have been left behind. Cerclage wires were placed for an apparent proximal femoral fracture. Postoperative X-rays demonstrated a large lucency tracking from the prior location of the distal interlocking screw to the calcar region. This finding made it evident that the broken screw had been retained in the nail and was dragged up the femur upon nail removal, causing a large gouge spanning the entire femur.

## Introduction

In femoral fracture fixation with a cephalomedullary nail, distal interlocking screw breakage, also known as autodynamization, is a well-described phenomenon [1]. Several factors can contribute to autodynamization including delayed fracture union, smaller screw diameter, the distance between the fracture and the most proximal interlocking screw, and the number of interlocking screws used [1]. The presence of a broken interlocking screw in patients who require the removal of their cephalomedullary nail, such as for conversion arthroplasty, presents a distinct challenge. Typically, the intact interlocking screw can be removed with a screwdriver through the same lateral incision used for its insertion. If the interlocking screw is broken, the medial screw fragment may be retained or removed, depending on whether the screw can be disengaged from the nail. We describe a unique complication relating to the retention of a broken distal interlocking screw during the removal of a long cephalomedullary nail for hip conversion arthroplasty.

## Case presentation

**Figure 1:**
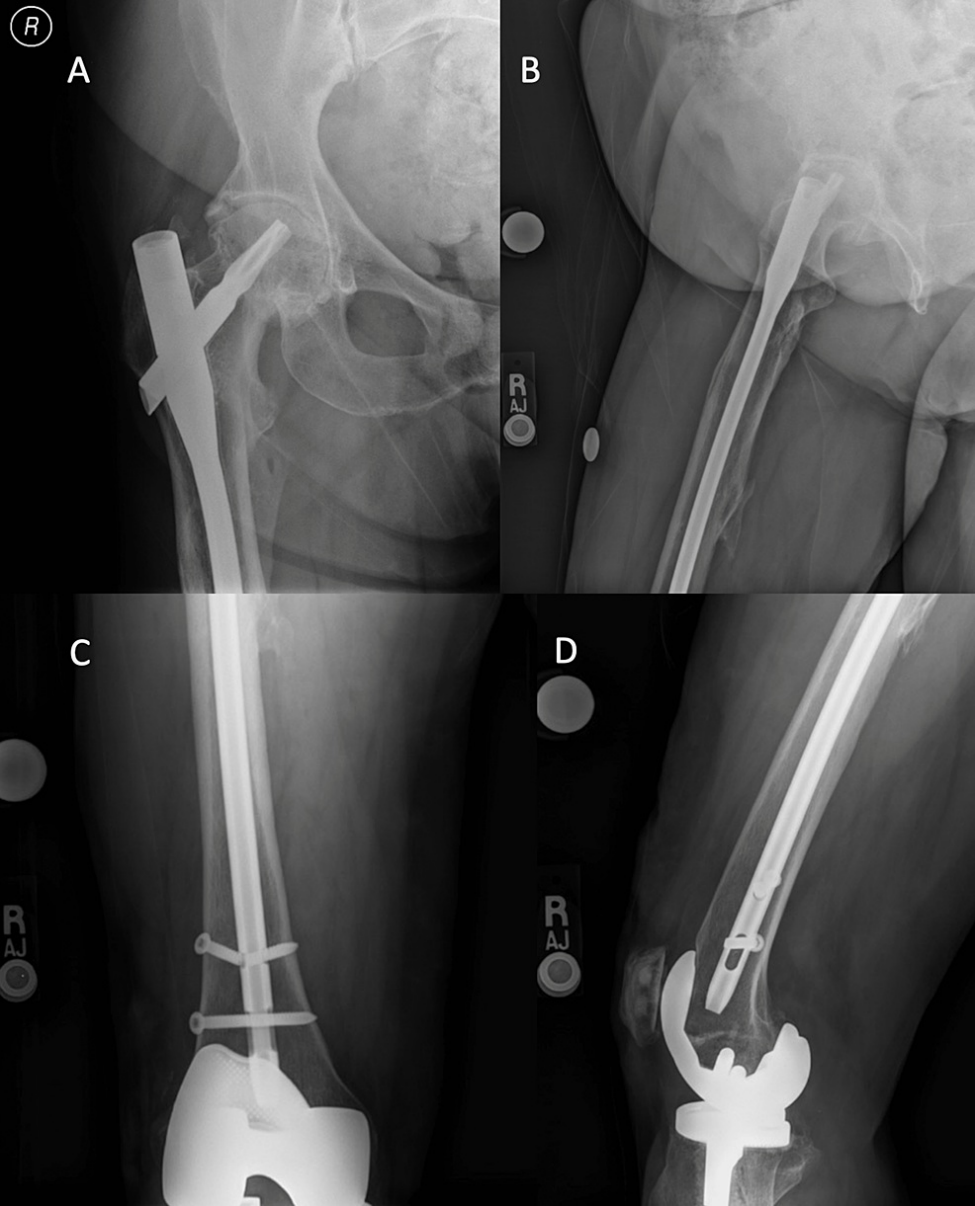
Preoperative radiographs Anteroposterior (A,C) and lateral (B,D) right femur radiographs demonstrating a healed subtrochanteric femur fracture with autodynamization of the distal interlocking screws and advanced right hip degenerative joint disease.

A 69-year-old female with a remote history of a right subtrochanteric femur fracture treated with a long cephalomedullary nail (Trochanteric Fixation Nail, Depuy Synthes, West Chester, PA) presented with symptomatic right hip osteoarthritis. Imaging demonstrated a healed fracture and autodynamization of the proximal-distal interlocking screw (Figure 1).

After a discussion of treatment options with the patient, she elected to proceed with conversion total hip arthroplasty. Using the patient’s existing incisions, a modified Hardinge approach was used to expose the hip joint. After the dislocation of the hip, it was reduced again, and attention was turned to the removal of the cephalomedullary nail. Through the proximal incision, the set screw was reversed, and the blade of the device was removed using the device-specific removal tools. Next, using the patient’s existing distal incisions (Trochanteric Fixation Nail, Depuy Synthes, West Chester, PA), the intact distal screw and the lateral fragment of the proximal screw were removed with a screwdriver. The nail extraction tool was engaged, and the nail was gently removed. Because the nail was able to be removed without resistance and no screw fragments were revealed upon the nail’s removal, it was assumed that the broken screw fragment had disengaged and was retained in the distal femur.

Next, the total hip arthroplasty was completed using a standard cementless acetabular component (Continuum, Zimmer Biomet, Warsaw, IN) and a fit-and-fill design proximally fitting a cementless femoral component (Trabecular metal stem, Zimmer Biomet, Warsaw, IN). During femoral component implantation, a proximal femoral fracture was noted. After further exposure of the fracture line, it appeared to track distally, necessitating the placement of two cerclage wires above and below the lesser trochanter. Finally, the femoral component was impacted into place with a firm endpoint and no apparent fracture displacement.

**Figure 2:**
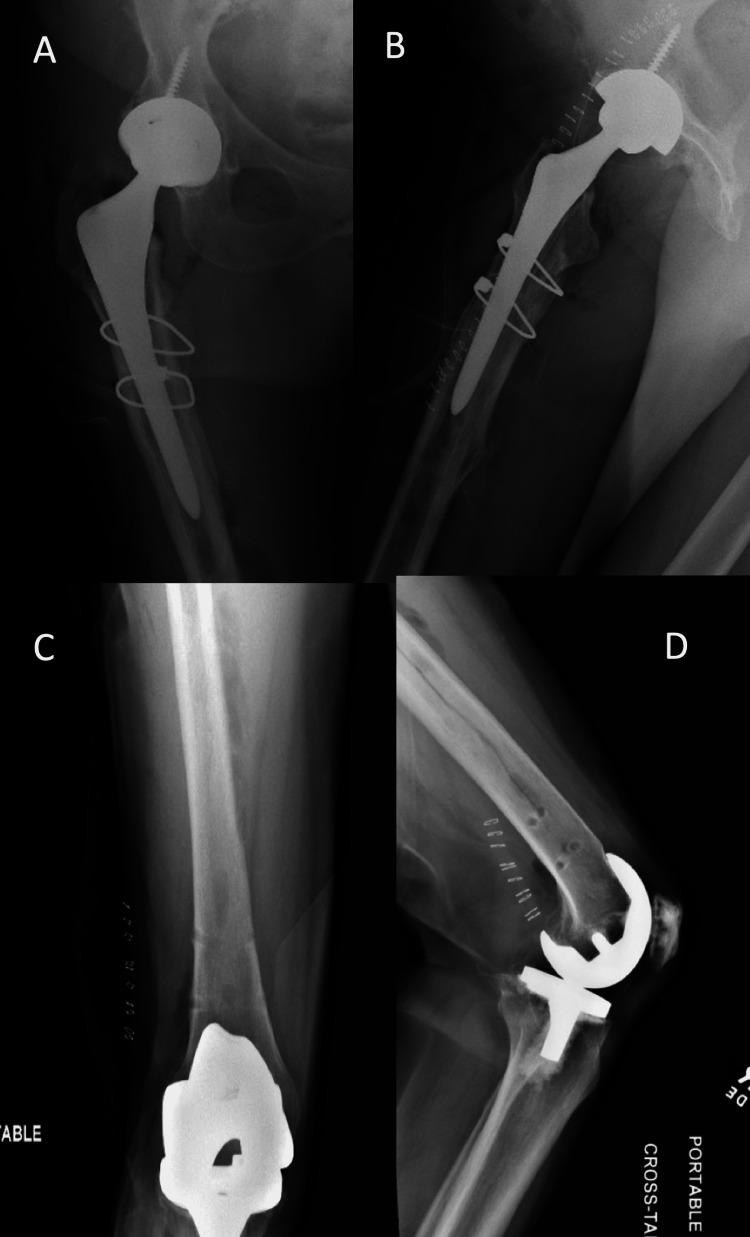
Immediate postoperative radiographs Anteroposterior (A,C) and lateral (B,D) immediate postoperative radiographs of the right hip and femur. There is a total hip arthroplasty secured with two cables. The distal interlocking screws have been removed, and there is a linear lucency seen advancing superiorly from the more proximal screw hole along the medial femoral shaft.

Postoperative X-rays demonstrated a total hip arthroplasty in the appropriate position with linear lucency tracking from the proximal screw hole to the calcar. In addition, the medial broken screw fragment was absent from the distal femoral region. With these findings, it was determined that the linear lucency was secondary to the medial aspect of the broken screw being retained within the nail and gouging the femoral canal as the nail was removed (Figure 2).

**Figure 3:**
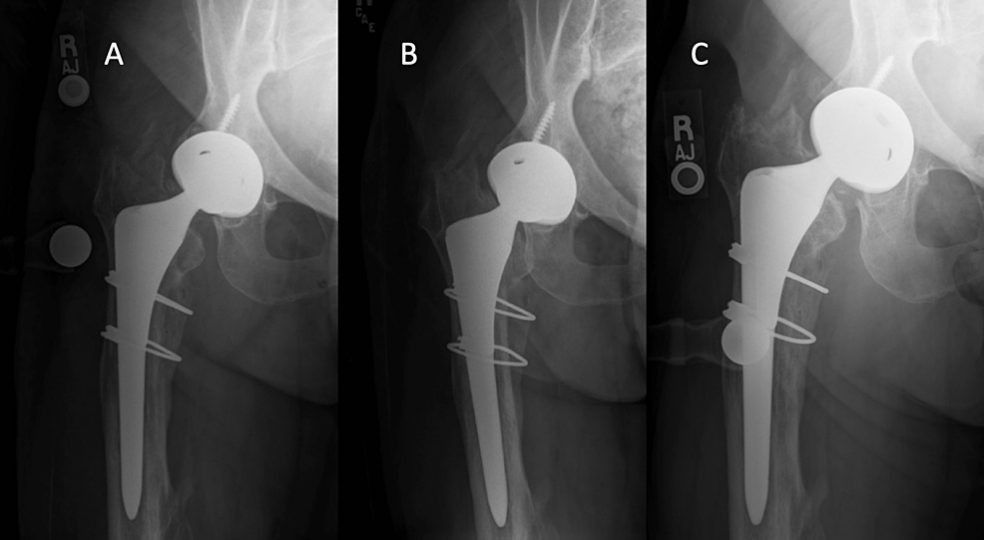
Follow-up postoperative radiographs Radiographs of the right hip taken at 4 weeks (A), 3 months (B), and 6 months (C) after surgery demonstrating stable implant subsidence without propagation of the intraoperative fracture and stability of the linear lucency.

The patient’s immediate postoperative course was uncomplicated, and the patient was made touch-down weight-bearing. At the four-week postoperative visit, the patient presented walking with a cane and admitted non-compliance with the weight-bearing restriction. Radiographs obtained at this time demonstrated femoral stem subsidence of approximately 1.5 cm with no change in the radiographic linear lucency. The potential risks and benefits of the surgical and non-surgical options to address the implant subsidence were discussed, and a collective decision was made to proceed with compliant touch-down weight-bearing with the goal of achieving ingrowth in the subsided position. At each subsequent follow-up appointment, the radiographs revealed no evidence of further subsidence or failed ingrowth (Figure 3).

**Figure 4:**
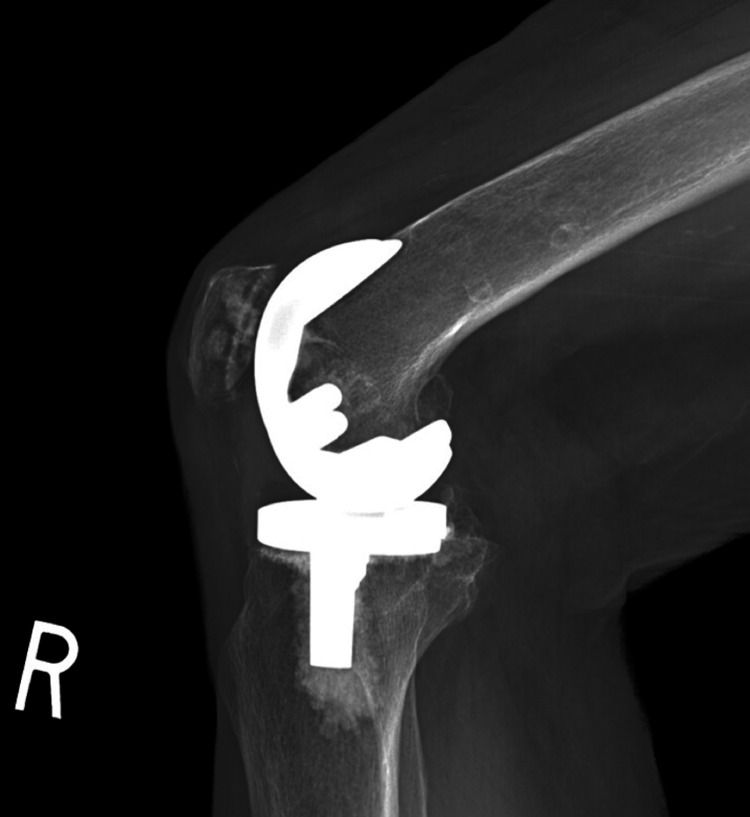
Lateral radiograph of the knee demonstrating absence of the linear lucency five years after surgery

Lateral radiographs of the knee taken five years after surgery revealed complete healing of the femoral gouge (Figure 4).

The patient ultimately had a successful outcome with a Short Form 12-point physical outcome score of 43.1 and a Veterans RAND 12-point outcome score of 43.4.

## Discussion

Hip conversion arthroplasty cases often pose substantial challenges related to hardware removal. Our case involved the removal of a cephalomedullary nail in the setting of a broken distal screw. Because the nail was removed without resistance, it was incorrectly assumed that the screw had disengaged from the nail and was retained in the distal femur. This resulted in an iatrogenic femoral gouge spanning the entire femur, which likely contributed to stem subsidence. In light of this complication, we recommend the removal of the broken medial fragment or fluoroscopic visualization of the distal screw prior to stem removal to confirm disengagement from the nail.

Several previous studies have described different techniques for broken screw removal [2]. Most methods involve retrieval of the fragment with a grasping tool through a medial incision [3]. Alternatively, if the medial fragment does not extend sufficiently through the far cortex, a Steinmann pin or guidewire can be used to "punch" the medial piece further into the far cortex prior to retrieval through a medial incision [2,4]. In order to avoid dissection of the medial femur, an alternative approach would be to use the "punch" technique to disengage the medial screw fragment from the nail to allow it to be retained rather than removed [3].

If the complication had been recognized intraoperatively, the surgeon would need to consider fixation of the gouge and femoral stem fixation. Since the gouge was located on the medial aspect of the femur, a typical lateral plate would not achieve the goal of securing the stabilization of the gouge. Placing a plate anteriorly would have helped achieve this secure fixation, but would not be recommended due to the added surgical trauma and potential complications of securing a plate in this location. As such, the gouge would have been best secured with multiple cerclage wires spanning the line of the gouge or multiple anteroposterior screws distal to the femoral stem combined with multiple cerclage wires at the level of the femoral stem. For femoral stem fixation, due to the length of the gouge, a long-stemmed femoral component would not have been able to bypass it. Femoral stem options that could have been considered include a short conical stem, a tapered metaphysically engaging stem (as was used in this case), or a cemented stem. Although a cemented stem may have diminished the risk of stem subsidence, we would still recommend limited weight-bearing in the initial postoperative period to protect the fixation of the gouge.

## Conclusions

With proper pre-operative planning and the use of intraoperative fluoroscopy, potential complications related to broken interlocking screws can be prevented in total hip conversion arthroplasty. There are inherent risks to the retention and removal of broken distal interlocking screws. Surgeons should address the broken screws preoperatively, even if they intend to leave the fragment in place. The authors recommend thorough preoperative planning and the use of intraoperative fluoroscopy during the removal of a cephalomedullary nail, as this would reveal retained screw fragments and prompt early intervention to prevent complications.
